# FAST: Fast, Free, Consistent, and Unsupervised Oligodendrocyte Segmentation and Tracking System

**DOI:** 10.1523/ENEURO.0025-24.2024

**Published:** 2025-02-07

**Authors:** Eunchan Bae, Gregory E. Perrin, Virgilio Gonzenbach, Jennifer L. Orthmann-Murphy, Russell T. Shinohara

**Affiliations:** ^1^Penn Statistics in Imaging and Visualization Center (PennSIVE), Department of Biostatistics, Epidemiology, and Informatics, Perelman School of Medicine, University of Pennsylvania, Philadelphia, Pennsylvania 19104; ^2^Department of Neurology, Perelman School of Medicine, University of Pennsylvania, Philadelphia, Pennsylvania 19104; ^3^Center for Biomedical Image Computing and Analytics, Department of Radiology, Perelman School of Medicine, University of Pennsylvania, Philadelphia, Pennsylvania 19104

**Keywords:** cell annotation, cell tracking, glia, in vivo Imaging, microscopy image processing, oligodendrocytes, two-photon imaging

## Abstract

To develop reparative therapies for neurological disorders like multiple sclerosis (MS), we need to better understand the physiology of loss and replacement of oligodendrocytes, the cells that make myelin and are the target of damage in MS. *In vivo* two-photon fluorescence microscopy allows direct visualization of oligodendrocytes in the intact brain of transgenic mouse models, promising a deeper understanding of the longitudinal dynamics of replacing oligodendrocytes after damage. However, the task of tracking the fate of individual oligodendrocytes requires extensive effort for manual annotation and is especially challenging in three-dimensional images. While several models exist for annotating cells in two-dimensional images, few models exist to annotate cells in three-dimensional images and even fewer are designed for tracking cells in longitudinal imaging. Notably, existing options often come with a substantial financial investment, being predominantly commercial or confined to proprietary software. Furthermore, the complexity of processes and myelin formed by individual oligodendrocytes can result in the failure of algorithms that are specifically designed for tracking cell bodies alone. Here, we propose a fast, free, consistent, and unsupervised beta-mixture oligodendrocyte segmentation system (FAST) that is written in open-source software, and can segment and track oligodendrocytes in three-dimensional images over time with minimal human input. We showed that the FAST model can segment and track oligodendrocytes similarly to a blinded human observer. Although FAST was developed to apply to our studies on oligodendrocytes, we anticipate that it can be modified to study four-dimensional *in vivo* data of any brain cell with associated complex processes.

## Significance Statement

We have developed “FAST: Fast, free, consistent, and unsupervised oligodendrocyte segmentation and tracking system” to solve our challenge of quantification of four-dimensional data acquired from longitudinal *in vivo* imaging. Although it was developed for oligodendrocytes, we will make the code entirely open source and user-friendly, and expect that it will be useful for segmentation for any cell body from a complex cell amenable to longitudinal *in vivo* imaging.

## Introduction

Oligodendrocytes are the myelinating cells of the central nervous system (CNS). Myelination of axons leads to faster, more efficient communication with other neurons, among other functions, and myelin loss contributes to debilitating functional deficits in multiple sclerosis (MS). The longitudinal study of demyelination *in vivo* using magnetic resonance imaging (MRI) (Oh et al., 2019) has traditionally focused on white matter regions, where myelin is dense and aligned. Unlike white matter, however, axons are variably myelinated in the cerebral cortex (Tomassy et al., 2014; Micheva et al., 2016), and cortical lesions in MS are not detected by standard MRI images. Recently, a novel *in vivo* two-photon fluorescence microscopy platform has been developed to visualize oligodendrogenesis and myelin sheath formation in cortical circuits at high resolution, so that their fate can be tracked over time (Hill et al., 2018; Hughes et al., 2018). This technology allows for the investigation of the loss and replacement of oligodendrocytes and myelination patterns in acquired demyelination models (Bacmeister et al., 2020; Orthmann-Murphy et al., 2020).

With the development of this new imaging method, there is a growing need for automated quantification and analysis of changes in oligodendrocyte fate. The analysis involves extracting the locations of oligodendrocyte cell bodies in three-dimensional images, and then defining the fate and/or location of these cell bodies over repeated imaging sessions. Currently, imaging scientists visually assess images to label and track the cell bodies over space and time. However, manual segmentation is costly, time-consuming and prone to inter- and intra- observer variability. Alternatively, longitudinal automated segmentation would provide an efficient, consistent, scalable and reproducible way to segment the cell bodies of oligodendrocytes. Here, we propose a fast, free, consistent, and unsupervised beta-mixture oligodendrocyte segmentation and tracking system (FAST) that can segment and track oligodendrocytes in longitudinal three-dimensional images.

Many tools have been developed for cell segmentation in images with recent advances in machine learning. Among freely available softwares, Cell-Profiler is widely used in biology, and can count the cell body in three-dimensional image and can track cell body in two-dimensional images, but not on three-dimensional images (Carpenter et al., 2006), limiting its use in two-photon image volumes. CellReg is a model to track neurons in calcium imaging (Sheintuch et al., 2017) and this model can track cell bodies in two-dimensional images, but not on three-dimensional images. OSLO is an unsupervised model to count oligodendrocyte progenitor cells in Z-stacked two-dimensional images, but it is not developed for tracking cells over time (Ma et al., 2018). Oligo-Track is a recently developed convolutional neural network (CNN) based model to track oligodendrocytes in longitudinal three-dimensional images (Xu et al., 2021). However, this model requires a modest number of training data with full annotation. Also, this model requires extensive computing power to run (for example, an RTX 2080 Ti GPU) and takes 45–55 min per image set. Furthermore, none of the previously published methods are designed to specifically address variation in the noise levels in images (for example, fluorescence intensity of oligodendrocyte cell body and processes change with cortical depth; Orthmann-Murphy et al., 2020). Imaris is a commercial image segmentation tool that can count and track cells in three-dimensional images (Miura et al., 2016). However, the cost for this tool is often financially burdensome for individual users. Additionally, users are not able to access or modify the source code to adapt to their experiment.

The novelty of the FAST model is threefold. First, we modeled the intensity value of the image with mixtures of two beta distributions. The beta distribution, unlike the normal distribution, is not symmetric around the mean and thus is sufficiently flexible to model the asymmetric intensity values observed in two-photon imaging. We fitted a mixture of two beta distributions to each two-dimensional images and binarized the microscopy image into background and region of interest (ROI). For the model fitting step, we adopted an expectation-maximization (EM) algorithm (Dempster et al., 1977). Second, we de-noised the images using a median filter while accounting for the morphology of oligodendrocytes and myelin sheaths. In our two-dimensional two-photon microscopy images, myelin sheaths have relatively low fluorescent signal intensity compared to oligodendrocyte somas, and for annotation purposes we consider them as anisotropic noise. The median filter is a non-linear filter that is shown to be effective in attenuating anisotropic noise (Qiu, 1996; Ng and Ma, 2006). In our analysis, we adopted a three-dimensional window since the morphology of oligodendrocytes is spherical in shape while the myelin sheath is linear. Third, we implemented a connected component labeling (CCL) algorithm to segment and track oligodendrocytes. CCL assigns a unique label to all voxels in a binary image based on spatial connectedness (He et al., 2017). Without a training step, which requires significant manual input, the CCL algorithm provided an automated, consistent and result that is consistent with manual segmentation.

## Materials and Methods

**Figure 1.**
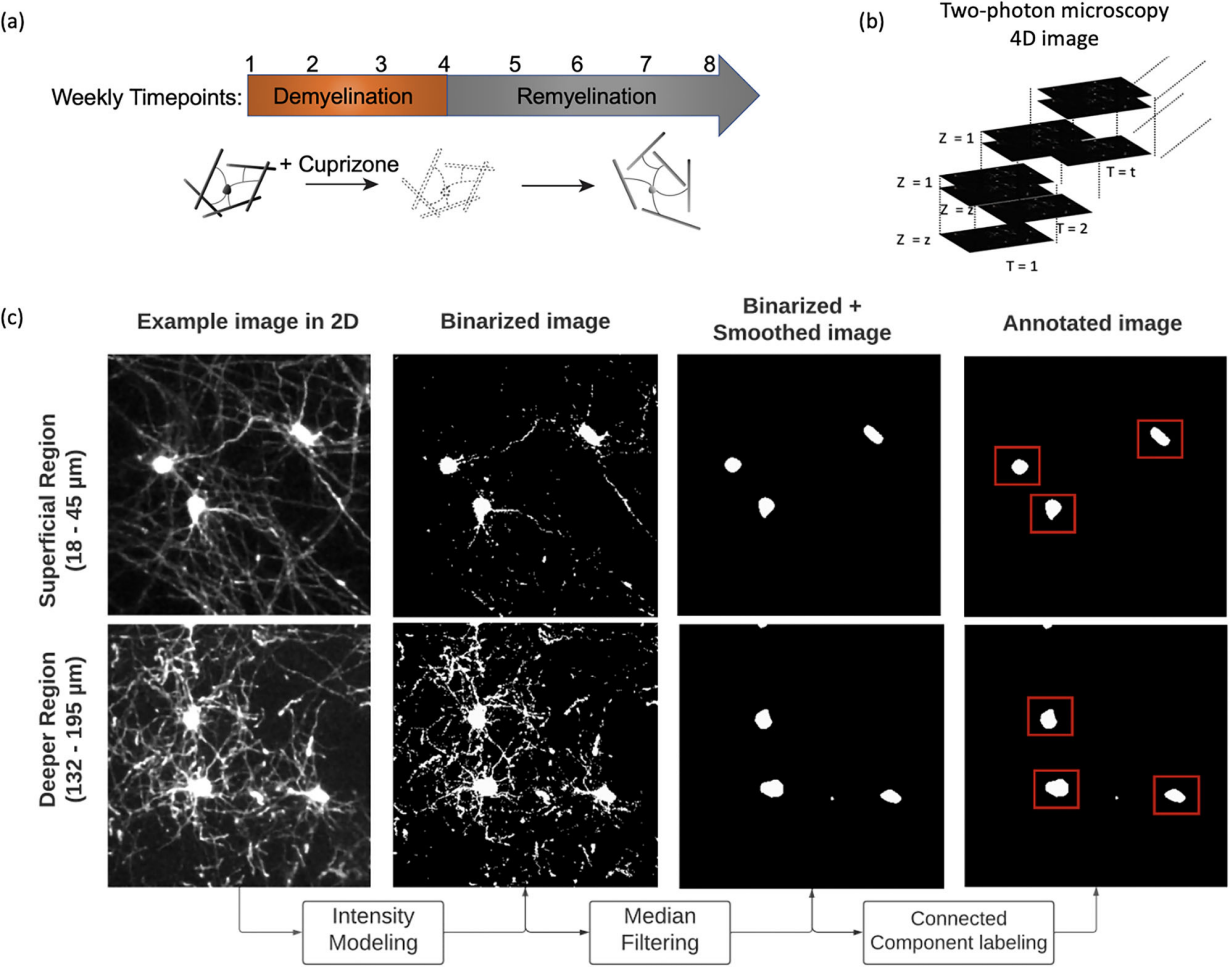
An illustration of the FAST model. ***a***, An illustration of the longitudinal course of loss (demyelination) and replacement (remyelination) of cortical oligodendrocytes as previously shown in Orthmann-Murphy et al. (2020) ***b***, An illustration of the raw, *in vivo* two-photon fluorescence microscopy four-dimensional images. ***c***, A schematic demonstrating the FAST model for a superficial (18 − 45 μm) and deeper (132 −195 μm) cortical region acquired by two-photon microscopy (for illustration purposes, a maximum intensity projection of each region of interest is shown). From longitudinally aligned four-dimensional images, intensity values from each two-dimensional image are modeled as a mixture of two beta distributions. From the binarized image, a median filter is applied to denoise the image. Then, connected component labeling (CCL) is applied to annotate the oligodendrocytes. The oligodendrocytes within the red rectangle represent the FAST model annotated oligodendrocytes.

**Figure 2.**
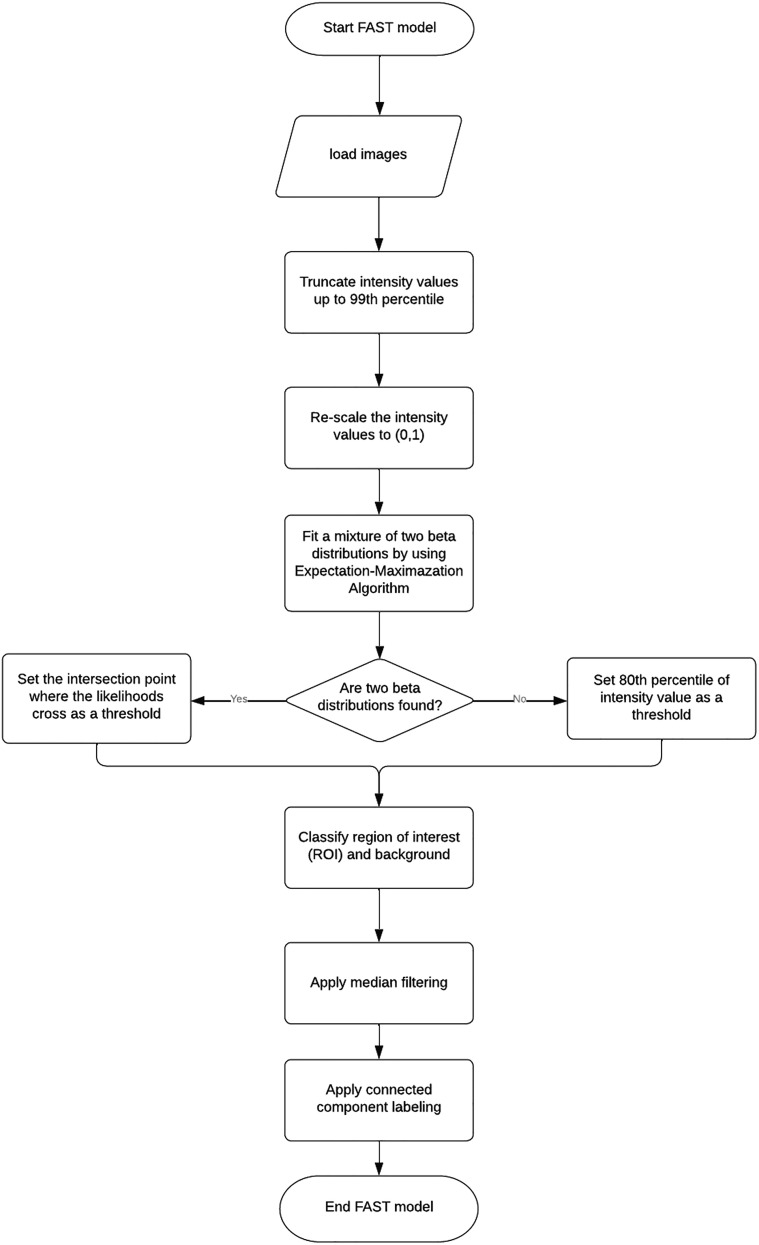
Pipeline of the FAST model. The FAST model takes longitudinally aligned images as input. With pre-determined parameters, the FAST model labels voxels under a threshold as background. After re-scaling the intensity value to [0,1], a mixture of two beta distributions is fit. If two beta distributions are fit, then set the intersection point as a threshold to label the region of interest (ROI) and background. If only one beta mixture component is detected, then voxels under a specified threshold are labeled as background, and those above the threshold are labeled as cell bodies. Then, a median filter is applied to denoise the ROI and oligodendrocytes are labeled using connected component labeling (CCL).

We developed an unsupervised segmentation system for annotating and tracking oligodendrocytes in three-dimensional images (location denoted by (*i*, *j*, *k*), where *i* = 1, …, *I*, *j* = 1, …, *J*, *k* = 1, …, *K*) across multiple timepoints (time-point denoted by *t*, where *t* = 1, …, *T*). An (a) illustration of the longitudinal course of loss (demyelination) and replacement (remyelination) of cortical oligodendrocytes and (b, c) schematic understanding the FAST model are described in Figure 1. We annotated cells using three main stages: (1) intensity modeling by fitting a mixture of beta distributions; (2) noise filtering by a median filter; and (3) annotation and registration by connected component labeling. The pipeline of the FAST model is presented in Figure 2. The FAST model was developed in the open source R software environment (version 4.0.2, R Core Team, 2021) and the software is available in https://github.com/PennSIVE/bossr. The FAST model was run on Linux machine with CPUs, but can be run on any operating systems including Windows and Macintosh. 

### Animals

Female and male adult mice were used for experiments and randomly assigned to experimental groups. All mice were healthy without obvious behavioral phenotypes. Generation and genotyping of MOBP-egfp (Gensat) and GLAST-CreER (Agarwal et al., 2017) x R26-lsl-tdTomato (JAX# 007914) (Agarwal et al., 2017; Hughes et al., 2018) as previously described. Mice were maintained on a 12 h light/dark cycle, housed in groups no larger than 5, and food and water were provided *ad libitum*. For cuprizone administration, at 9 to 11 weeks of age, male and female MOBP-egfp mice were fed a diet of milled, irradiated 18% protein rodent diet (Teklad Global) alone (control) or supplemented with 0.2% w/w bis(cyclohexanone) oxaldihydrazone (Cuprizone, Sigma-Aldrich) in custom gravity-fed food dispensers for 3 to 6 weeks. Both control and experimental condition mice were returned to regular pellet diet during the recovery period as previously reported (Orthmann-Murphy et al., 2020). All animal experiments were performed in strict accordance with protocols approved by the Animal Care and Use Committee at the Johns Hopkins University.

### Cranial windows and *in vivo* two-photon microscopy

As previously described (Orthmann-Murphy et al., 2020), we placed cranial windows over the somatosensory cortex of 7–10 week old adult mice. Two weeks after craniotomy, we acquired a “baseline” image using a Zeiss LSM 710 microscope equipped with a GaAsP detector using a mode-locked Ti:sapphire laser (Coherent Ultra) tuned to 920 nm. During imaging sessions, mice were anesthetized with isoflurane and immobilized by attaching the head plate to a custom stage. Vascular landmarks were identified in order to image the same cortical area over repeating sessions. Image stacks were acquired with a Zeiss 20x water immersion objectives with depth to cortical layer 4 relative to the pial surface, at baseline and then the same region every one to 7 d, for up to 15 weeks.

### Image acquisition and preprocessing

For oligodendrocyte annotation, we re-used previously reported data from eight longitudinal image stacks from MOBP-egfp mice from both male and female mice (Orthmann-Murphy et al., 2020). Image stacks were registered using the FIJI plugin “Correct 3D Drift” (Parslow et al., 2014), from *n* = 3 cuprizone-treated mice and *n* = 2 control mice. There were two regions each from two of the cuprizone-treated mice, one region from one cuprizone-treated mouse, two regions from one control mouse and one region from the other control mouse. The size of the images were 425 μm × 425 μm × 550 μm and measured in 1024 × 1024 × 190 voxels for analysis. Images were obtained every 1–2 weeks and followed through 10 weeks (9 timepoints) for cuprizone-treated mice and 9 weeks (8 timepoints) for control mice.

For astrocyte annotation, we used three longitudinal image stacks that were similarly acquired as above and processed for two control transgenic mice (GLAST-CreER, Agarwal et al., 2017 x R26-lsl-tdTomato (JAX# 007914) x MOBP-egfp).

### Intensity modeling

*In vivo* two-photon fluorescence microscopy allows visualization of oligodendrocytes with high resolution. However, the fluorescence intensity and contrast deteriorates with cortical depth. To account for heterogeneous intensity across cortical depth, we parameterized the intensity of each slice as a two-dimensional image.

The observed intensity of the original images was bounded in [0,255]. We first re-scaled the intensities to [0,1]. Then, we truncated up all pixels below the 99th percentile as most of the pixels were part of the image background. As the support of the intensity is bounded, we binarized the two-dimensional images by fitting a mixture of two beta distributions.

Let Y_*i*,*j*,*k*,*t*_ indicate the random variable of intensity value located at {*i*, *j*, *k*} and time-point *t* and **Y**_*i*,*j*_ indicate the collection of image intensity values in two-dimensional (*i*, *j*) image for fixed *k* and *t* with probability density function *f*.

We modeled Y_*i*,*j*,*k*,*t*_ (hereafter, abbreviated as Y) as coming from one of two different beta distributions *f*_0_, *f*_1_ with proportions *p*_0_ and *p*_1_. Let Z_*i*,*j*,*k*,*t*_ (hereafter, abbreviated as Z) indicate the latent variables representing the mixture component of Y such that P(Z = 0) = *p*_0_ and P(Z = 1) = *p*_1_ = 1 − *p*_0_ where Z ∈ {0, 1} at {*i*, *j*, *k*} and time-point *t* and **Z**_*i*,*j*_ indicate the collection of latent variables in two-dimensional (*i*, *j*) image for fixed *k* and *t*. Define a random variable Y conditional on the latent variable Z such that Y ∼ *f*_0_ if Z = 0 and Y ∼ *f*_1_ if Z = 1. Then, the conditional density *f*(Y | Z = *z*) = *f*_*z*_ is the beta distribution for the *z*th class. The joint probability density function of Z and Y is then given by *f*(Z = *z*, Y = *y*) = *p*_*z*_ *f*_*z*_(*y*). The marginal density of **Y**_*i*,*j*_ is given as follows:

$$f(Y_{i,j})=\prod_{i=1}^{I}\prod_{\;j=1}^{J}\{f(\text{Y}=y)\}=\prod_{i=1}^{I}\prod_{\;j=1}^{J}\left\{\sum_{z=0}^{1}f(\text{Z}=z,\text{Y}=y)\right\}=\prod_{i=1}^{I}\prod_{\;j=1}^{J}\prod_{z=0}^{1}\{p_{z}f_{z}(y){\}}^{I(\text{Z}=z)},$$

where

$$f_{z}(y)=f(y\;|\;\alpha_{z},\beta_{z})=\frac{y^{\alpha_{z}-1}(1-y{)}^{\beta_{z}-1}}{\text{B}(\alpha_{z},\beta_{z})}$$

for *z* = 0, 1 and $\text{B}(\alpha ,\beta )=\frac{\Gamma (\alpha )\Gamma (\beta )}{\Gamma (\alpha +\beta )}$, where Γ( · ) is the gamma function. Our goal is to estimate unknown parameters, *α*_0_, *α*_1_, *β*_0_, *β*_1_ and *p*_0_, to maximize the *f*(**Y**). We estimated these unknown parameters by implementing an expectation maximization (EM) algorithm (Dempster et al., 1977).

Let *θ* = (*α*_0_, *α*_1_, *β*_0_, *β*_1_, *p*_0_) indicate the vector of unknown parameters that we want to estimate and *θ*^(*r*)^ indicate the current value of parameters, *θ*^(*r*+1)^ indicate the updated parameters. Let *L*(*θ* | **Y**_*i*,*j*_, **Z**_*i*,*j*_) and *l*(*θ* | **Y**_*i*,*j*_, **Z**_*i*,*j*_) indicate the likelihood and log-likelihood function for complete data. We can formulate the likelihood as follows:

$$L(\theta \;|\;Y_{i,j})=f(Y_{i,j}\;|\;\theta )=\int f(Y_{i,j},Z_{i,j}\;|\;\theta )\;dZ_{i,j}=\int f(Z_{i,j}\;|\;\theta ,Y_{i,j})f(Y_{i,j}\;|\;\theta )\;dZ_{i,j}.$$

As we do not observe the **Z**_*i*,*j*_, we implemented an EM algorithm to find the maximum likelihood estimator $\hat{\theta }$ by iterating E- and M-steps as follows:

#### E-step

Define *Q*(*θ* | *θ*^(*r*)^) as the expected value of the log-likelihood of *θ* with respect to the conditional distribution of **Z**_*i*,*j*_ given **Y**_*i*,*j*_ and current parameters *θ*^(*r*)^.

$$Q(\theta \;|\;\theta^{\left(r\right)})={\text{E}}_{Z_{i,j}\;|\;Y_{i,j},\theta^{\left(r\right)}}[l(\theta \;|\;Y_{i,j},Z_{i,j})].$$

In E-step, we calculate *Q*(*θ* | *θ*^(*r*)^) as follows:

$$\begin{array}{rl} & {\text{E}}_{Z_{i,j}\;|\;Y_{i,j},\theta^{\left(r\right)}}[l(\theta \;|\;Y_{i,j},Z_{i,j})]\\ & \;={\text{E}}_{Z_{i,j}\;|\;Y_{i,j},\theta^{\left(r\right)}}\left[\sum_{i=1}^{I}\sum_{\;j=1}^{J}\sum_{z=0}^{1}I(Z=z)\{\log(p_{z})+\log\;(f_{z}(y\;|\;Z=z,\theta^{\left(r\right)}))\}\right]\\ & \;=\sum_{i=1}^{I}\sum_{\;j=1}^{J}\sum_{z=0}^{1}\{{\text{E}}_{Z_{i,j}\;|\;Y_{i,j},\theta^{\left(r\right)}}[I(Z=z)]\}\{\log\;(p_{z})+\log\;(f_{z}(y\;|\;Z=z,\theta^{\left(r\right)}))\}. \end{array}$$

Note that

$$\begin{array}{rl} {\text{E}}_{Z_{i,j}\;|\;Y_{i,j},\theta^{\left(r\right)}}[I(\text{Z}=z)]=f(\text{Z}=z\;|\;\text{Y}=y,\theta^{\left(r\right)}) & =\frac{\;f_{z}(\text{Y}=y\;|\;\text{Z}=z,\theta^{\left(r\right)})\cdot f(Z=z\;|\;\theta^{\left(r\right)})}{f(\text{Y}=y\;|\;\theta^{\left(r\right)})}\\ & =\frac{\;f_{z}(\text{Y}=y\;|\;\text{Z}=z,\theta^{\left(r\right)})\cdot p_{z}}{\sum_{z=0}^{1}f_{z}(\text{Y}=y\;|\;\text{Z}=z,\theta^{\left(r\right)})\cdot p_{z}}. \end{array}$$

#### M-step

In the M-step, we update the parameters *θ*^(*r*+1)^ by maximizing *Q*(*θ* | *θ*^(*r*)^) such that

$$\theta^{\left(r+1\right)}={\text{argmax}}_{\theta }Q(\theta \;|\;\theta^{\left(r\right)}).$$

There is no closed form solution to maximize *Q*(*θ* | *θ*^(*r*)^), so we used a numerical approximation function, *stats* : : *optim*(), in R to find the maximum likelihood estimators.

For initialization, we randomly assigned *Y* to belong either *Z* = 0 or *Z* = 1, as has been found to be efficient (Biernacki et al., 2003). If the EM algorithm converges with two beta distributions, then we retrieved the intersection point where the likelihoods cross and used this for thresholding. If the EM algorithm converges with one beta distribution, then we retrieved the 80th percentile of pixel intensities and used this for thresholding. Note that this 80th percentile value is flexible and the user can determine the optimal percentile based on image quality. Values above the threshold were labeled as region of interest (ROI) and values below this threshold were labeled as background.

Fitting a mixture model took less than 20 s per two-dimensional image with dimension [1024 × 1024]. In our analysis, each data set has a dimension of [1024 × 1024 × 190 × 9] or [1024 × 1024 × 190 × 10] and took around 10 h on a single core run serially. However, this step can be parallelized and computing time can be significantly reduced (Amdahl, 1967).

### Noise filtering

Oligodendrocytes have complex processes that extend from the cell body to interact with axons and form myelin sheaths; however, these processes are ancillary to cell body counting. We considered myelin sheaths in microscopy images as impulsive noise, which has small bright voxels in dark regions. Compared to linear filters (e.g., Gaussian filter and mean filter), median filters do not produce image blurring which is desired because image is already binarized to zero and one (George et al., 2018). We stacked the two-dimensional images that were binarized in **Intensity Modeling** and applied the median filter (Huang et al., 1979).

Let ${\text{Ẑ}}_{i,j,k,t}$ indicate the fitted latent variable of Z_*i*,*j*,*k*,*t*_ by intensity modeling in **Intensity Modeling** and let $N({\text{Ẑ}}_{i,j,k,t})$ indicate the collection of neighboring intensity values of ${\text{Ẑ}}_{i,j,k,t}$. The median filter procedure calculates the median value of the vector $N({\text{Ẑ}}_{i,j,k,t})$, ${\text{Ẑ}}_{i,j,k,t}^{*}$.

$${\text{Ẑ}}_{i,j,k,t}^{*}=\text{median}(N({\text{Ẑ}}_{i,j,k,t})).$$

Depending on the size of the cell body of oligodendrocytes, window size of median filter can be optimized. We used the window size of [11,11,3] for fixed *t* as the likelihood of detecting fluorescence signal in myelin sheaths is reduced with cortical depth while the cell body are more isotropic. Note that this size is flexible and user can choose the size based on the size of the oligodendrocytes and acquired image resolution. Also, this step does not exclude cell bodies based on their size; cell body size filtering is performed after annotation.

For computation, median filtering took less than 5 min with dimension [1024 × 1024 × 190] in single core and may take 50 min to filter full longitudinal three-dimensional image with dimension [1024 × 1024 × 190 × 10]. However, this step again could benefit from parallelization.

### Cell annotation, registration, and post-processing

In graph theory, a connected component is defined as a subgraph in which every two nodes are connected to each other by edges, but not connected to additional nodes in the supergraph. Let graph *G*_*i*_ = {*V*_*i*_, *E*_*i*_} indicate the *i*th four-dimensional graph with the node set *V*_*i*_ and edge set *E*_*i*_. From binarized, median-filtered intensity value ${\text{Ẑ}}_{i,j,k,t}^{*}$ located at {*i*, *j*, *k*} and time-point *t*, voxel {*i*, *j*, *k*, *t*} is considered node where ${\text{Ẑ}}_{i,j,k,t}^{*}$ = 1 while the tracts that connect the nodes of neighboring nodes are considered as edges.

To define the edges in four-dimensional images, we defined the neighborhood as the kernel size [3,3,3,3] such that only elements next to the ${\text{Ẑ}}_{i,j,k,t}^{*}$ is considered as neighborhood. The resulting neighboring value, $N^{*}({\text{Ẑ}}_{i,j,k,t}^{*})$, is $\left\{{\text{Ẑ}}_{i-1,j-1,k-1,t-1}^{*},{\text{Ẑ}}_{i,j-1,k-1,t-1}^{*},\ldots ,{\text{Ẑ}}_{i+1,j+1,k+1,t+1}^{*}\right\}$. Then, we determined the number of connected components **G** = {*G*_1_, *G*_2_, …, *G*_*i*_} in four-dimensional images (Dillencourt et al., 1992). For image sets with reduced signal-to-noise quality, larger kernel size can be used to accommodate the less spatially connected oligodendrocytes.

We expect to have a high signal-to-noise image throughout the experiment so that connected component labeling algorithm can form a subgraph over timepoints. However, if an interim image has low signal-to-noise (minimal detection of emitted signal), subgraphs are divided and therefore the FAST model reports two cells, one lost cell at the time-point of the low signal-to-noise image time-point and one new cell at the following time-point. To account for variations in image clarity over time, we treated the surrounding elements near {*i*, *j*, *k*} at each time-point *t* as belonging to the same group. This neighborhood kernel (representing the distance at which split cells are still considered part of the same cell, despite being split due to low image resolution) is then optimized. Once we post-process the images, we removed any smaller, isolated clusters from the overall structure. We filtered out the connected components in **G** that have node sets with fewer than 30 nodes. This enabled the algorithm to keep the cell body while reducing noise. This number is also a tuning parameter that is flexible, and larger or small size can be used based on the relative size of oligodendrocytes. Since the operation is sequential on four-dimensional array, parallelization is not available in this step.

#### Code accessibility

The code/software described in the paper is freely available online at https://github.com/PennSIVE/bossr. The code is available as Extended Data.

#### Verification of FAST cell fate output

To confirm the utility of the FAST model output, we developed a partially automated visual verification step. Using custom MATLAB code and FIJI, TIFF hyperstacks with FAST-labeled cells were parsed into individual stacks for each time point. 3D-corrected stacks were cropped to center the location of each FAST-identified cell, and then we manually reviewed each cell location, presence or absence within stacks at each time-point and compared to cell location label provided by the FAST model.

## Results

We applied the FAST model to longitudinal, *in vivo* two-photon fluorescence microscopy images to visualize, annotate and track the fate of individual cortical oligodendrocytes and astrocytes over time in adult transgenic mice.

**Figure 3.**
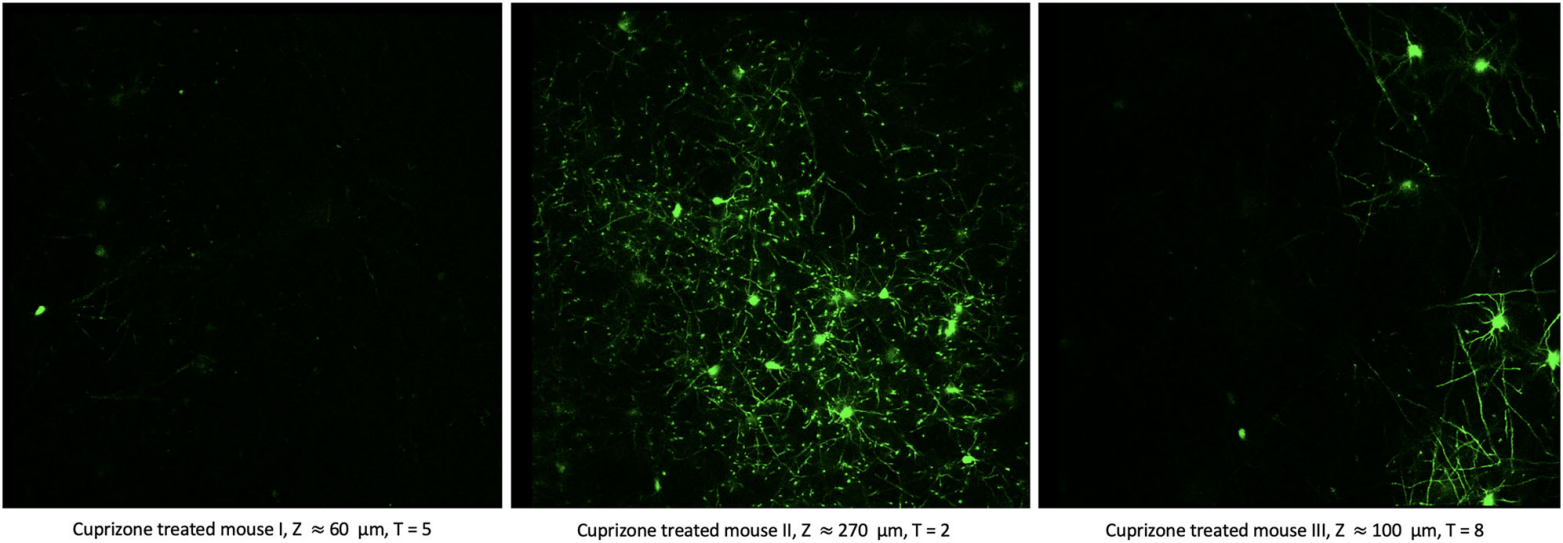
Images with 80th percentile intensity value threshold. Examples of images where the 80th percentile of intensity value is used as a threshold due to the impulsive noise with maximum intensity are provided. Each image is from a cuprizone-treated mouse and is chosen to illustrate cases where the two beta mixture components are not detected, and the 80th percentile of intensity value is used as the threshold. There was no association found between under- or over-annotation and the choice of threshold method.

For visualization of oligodendrocytes, transgenic mice that express enhanced green fluorescent protein (GFP) under control of the promoter for myelin-associated oligodendrocyte basic protein (MOBP) mice were used for imaging. To determine the fate of individual oligodendrocytes in healthy versus demyelinated cortex, young adult MOBP-egfp mice (age 8–12 weeks) were fed chow mixed with 0.2% cuprizone, a copper chelator that induces apoptosis of oligodendrocytes, or sham, for 3 weeks and then allowed to recover for at least 5 weeks. Cranial windows were placed over the somatosensory cortex, and the same region was monitored over the course of cuprizone-treatment and recovery. Throughout this article, five regions from three mice treated with cuprizone and three regions from two control mice were used to track oligodendrocytes. Details of imaging protocols are described in Orthmann-Murphy et al. (2020). We excluded one time-point from control mice as there was insufficient signal-to-noise to consistently identify cell bodies with the FAST model. The impact of including this image on the output of the FAST model is addressed in the **Discussion**. Occasionally, only one of two beta mixture components is detected due to the extremely low signal-to-noise (where no cells exist) or impulsive noise with maximum intensity (where cells are visible). Examples of images where the 80th percentile of intensity value is used as a threshold are presented in Figure 3.

To evaluate the performance of the FAST model, we compared it with annotations conducted previously by blinded human observers as previously reported for these images (Orthmann-Murphy et al., 2020). For oligodendrocytes, the total number of oligodendrocytes in a region, as well as the number of new oligodendrocytes and lost oligodendrocytes at later time-points, were annotated. To account for correlation across the multiple time-points, we fitted a linear mixed effect model with the number of annotated oligodendrocytes by blinded human observers as the outcome, the number of annotations by the FAST model as a fixed effect, and each image set as a random effect. An estimated coefficient greater than one implies the under-annotation whereas an estimated coefficient less than one implies the over-annotation. The model was fit and fixed effects as well as the profile likelihood-based confidence interval (CI) were calculated using *lmerTest* : : *lmer*() in R (Kuznetsova et al., 2017).

### Oligodendrocyte annotation results of the FAST model across timepoints

**Figure 4.**
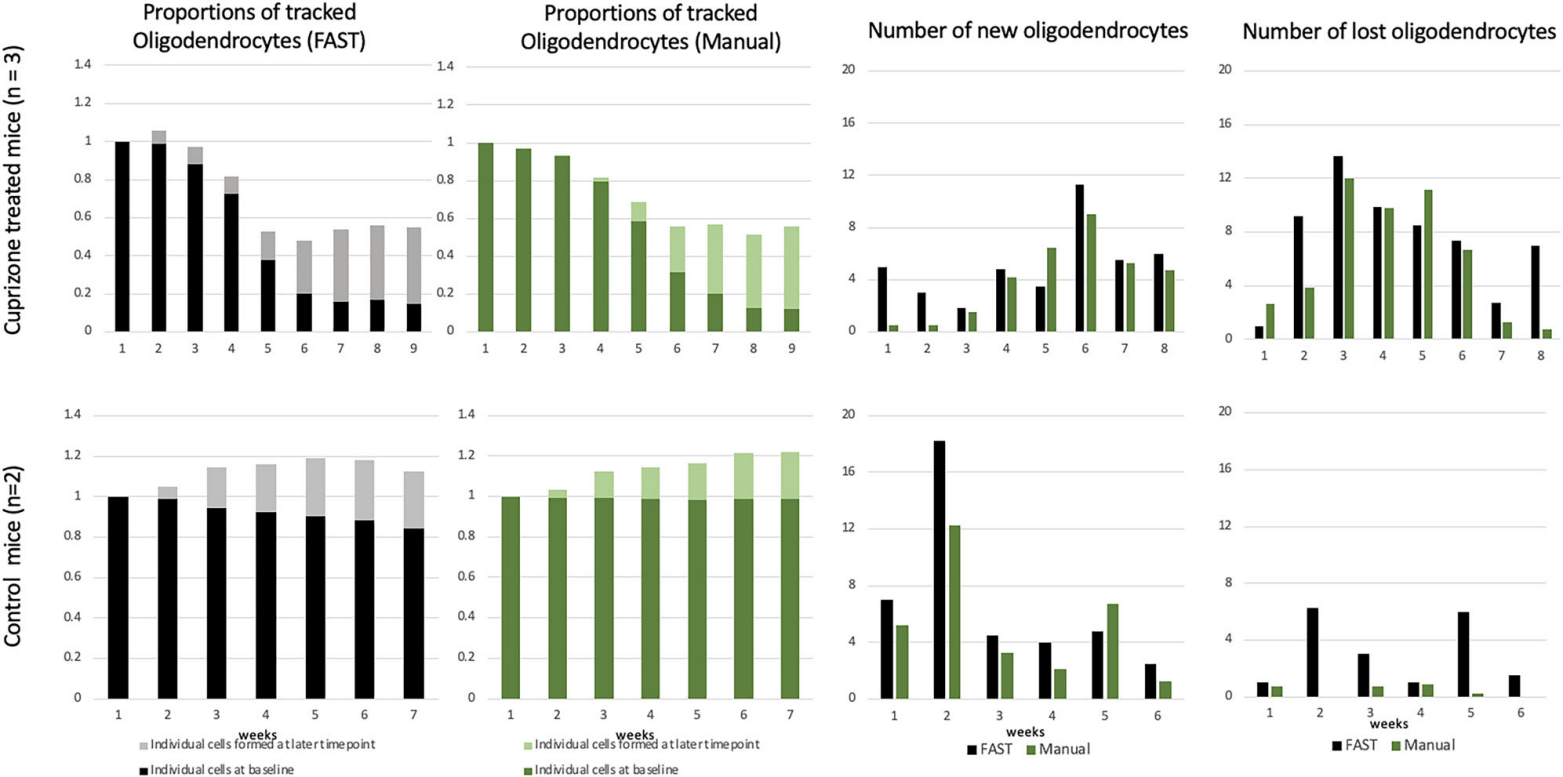
Results of the FAST model across timepoints. The first row shows the result of the FAST model when applied in cuprizone-treated mice (5 image sets from *n* = 3 mice). The second row shows the result of the FAST model when applied in control mice (3 image sets from *n* = 2 mice). The first/second column shows the proportion of the average number of tracked oligodendrocytes by the FAST model/blinded human observer compared to the week 1. The third column shows the average number of newly formed oligodendrocytes at each time-point. Each time-point represents the number of new oligodendrocytes observed at its next time-point. The fourth column shows the average number of lost oligodendrocytes at each time-point. Each time-point represents the number of lost oligodendrocytes observed at its next time-point.

**Figure 5.**
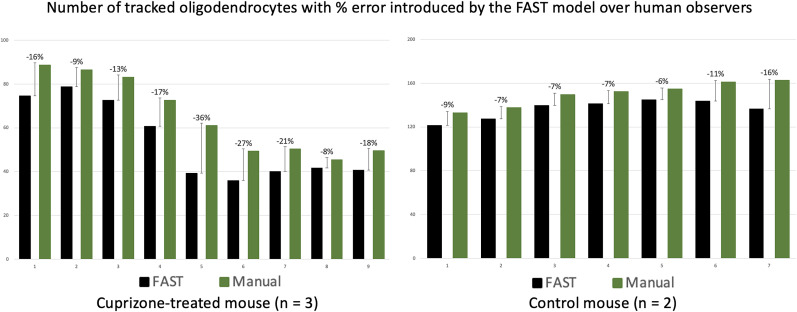
Results of the FAST model versus human observers across timepoints, measured by the total number. The bar graphs on the left display the results when evaluated on cuprizone-treated mice (5 image sets from *n* = 3 mice). The bar graphs on the right display the results when evaluated on the control mice (3 images sets from *n* = 2 mice). The error bars represent the % error introduced by the FAST model over human observers.

**Figure 6.**
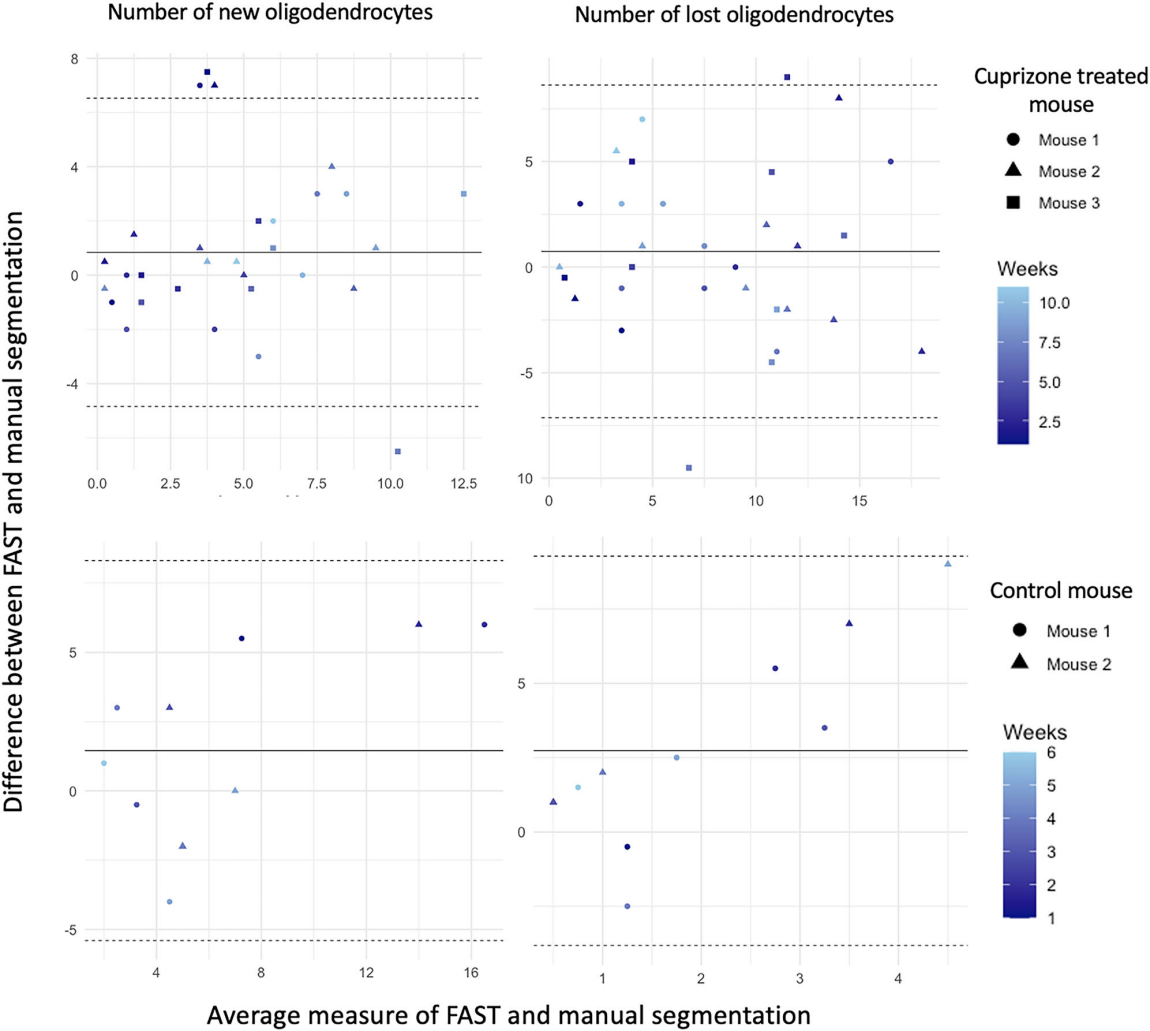
Bland–Altman (BA) plot for concordance evaluation. The BA plot of the new oligodendrocytes (first column) and lost oligodendrocytes (second column) are presented. *Y*-axis indicates the difference between FAST and manual segmentation and *X*-axis indicates the average measure of FAST and manual segmentation. A wider spread of points around the mean difference line or deviation from the zero-mean line suggest poorer agreement between the two methods. A tighter cluster of points around the mean difference line indicate better agreement. Straight bold line in BA plot indicates the mean difference and dashed line indicates the mean difference plus and minus 1.96 times the standard deviation of the differences for 95% CI under the null hypothesis that the mean differences are normally distributed.

We evaluated the FAST model using annotations conducted by blinded human observers as the gold standard. As shown in Figure 4, the bar graphs represent the proportion of the average number of oligodendrocytes in a 425 μm × 425 μm × 300 μm volume superficial region of somatosensory cortex compared to the baseline (week 1, 100%) for 3 mice treated with cuprizone and 2 control mice, as well as the average number of new/lost oligodendrocytes annotated by the FAST model and the blinded human observer. Figure 5 shows the number of tracked oligodendrocytes with % error introduced by the FAST model over the human observers. The Bland-Altman (BA) plots are also presented in Figure 6 to assess the agreement between two quantitative methods of measurement (Giavarina, 2015). The BA plot is a graphical method used to assess the agreement between two different measurement techniques or observers. A wider spread of points around the mean difference line or deviation from the zero-mean line suggest poorer agreement between the two methods. Conversely, a tighter cluster of points around the mean difference line indicate better agreement. The limits of agreement are often calculated as the mean difference ±1.96 times the standard deviation of the differences for 95% CI under the null hypothesis that the mean differences are normally distributed.

For images from cuprizone-treated mice, the FAST model under-annotated the number of oligodendrocytes to the blinded human observer compared to its baseline. The estimated regression coefficient of the FAST model on blinded human observer was 1.22 (95% CI: 0.93–1.51), indicating a 22% under-annotation of the number of oligodendrocytes after adjusting the multiple time-points. The FAST model over-annotated the number of new oligodendrocytes compared with the blinded human observer, but the difference was minimal (mean absolute difference: 1.83). Similarly, the FAST model over-annotated the number of lost oligodendrocytes over time-points, but the difference was also minimal (mean absolute difference: 2.45). Both the FAST model and the blinded human observer reported the maximum number of new/lost oligodendrocytes at the same time-point. Both reported the maximum number of new oligodendrocytes at the sixth time-point (corresponding to 1 week recovery) and the majority oligodendrocytes were lost between the third and sixth timepoints (corresponding to 2 weeks of cuprizone treatment through 2 weeks of recovery). BA plots (in the first row) show the satisfactory agreement between the FAST model and the blinded human observer as almost all data points are within the 1.96 times the standard deviance of the differences. BA plots of the new/lost oligodendrocytes show the unbiased measurement of the FAST model compared to the manual annotation.

For images from control mice, the FAST model under-annotated the number of oligodendrocytes to the blinded human observer compared to its baseline. The estimated regression coefficient of the FAST model on blinded human observer was 1.14 (95% CI: 0.83 to 1.46), indicating a 12% under-annotation of the number of oligodendrocytes after adjusting the multiple time-points. The FAST model over-annotated the number of new oligodendrocytes over time-points, but the difference was minimal (mean absolute difference: 2.35). The FAST model over-annotated the number of lost oligodendrocytes over time-points, but the difference was minimal (mean absolute difference 2.69). BA plots (in second row) show satisfactory agreement between the FAST model and the blinded human observer as all data points are within the 1.96 times the standard deviance of the differences. BA plots (in second row) show unbiased agreement between the FAST model and the blinded human observer as all data points are within the 1.96 times the standard deviance of the differences.

### FAST model annotation of oligodendrocytes across timepoints, stratified by the depth of somatosensory cortex

**Figure 7.**
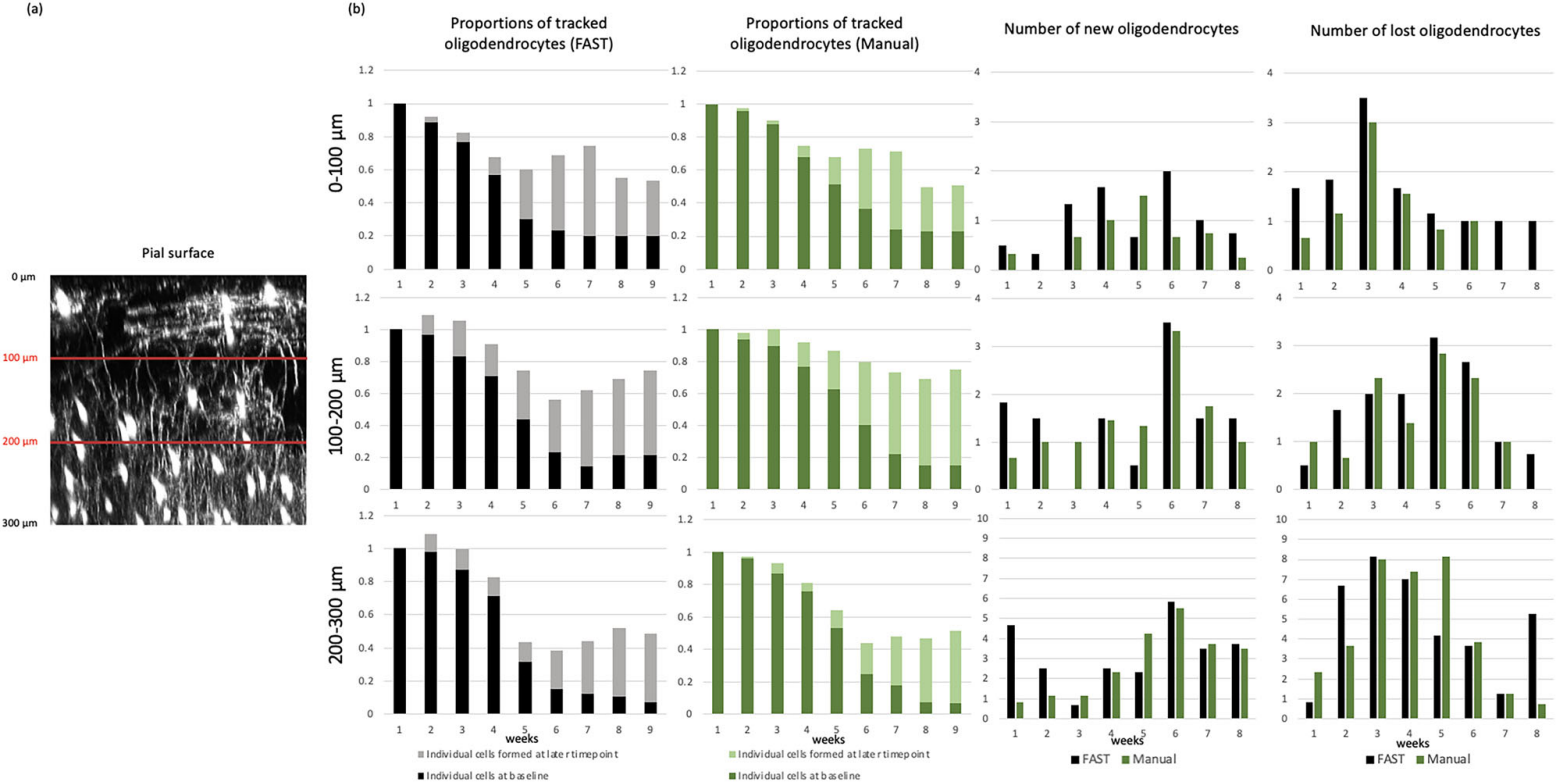
Results of the FAST model across time-points, stratified by the depth of somatosensory cortex. ***a***, An example of a cortical region containing GFP+ oligodendrocytes, shown as a maximum intensity Y-projection, separated by red lines at intervals of 100 μm relative to the pial surface. ***b***, Results of the FAST model across time-points stratified by depth in somatosensory cortex for cuprizone-treated mice (*n* = 3). The rows indicate the 0 − 100 μm, 100 − 200 μm, and 200 − 300 μm depth regions. The first column shows the proportion of the average number of tracked oligodendrocytes by the FAST model compared to the week 1. The second column shows the proportion of the average number of tracked oligodendrocytes by blinded human observer compared to the week 1. The third column shows the average number of newly formed oligodendrocytes. The rightmost column shows the average number of lost oligodendrocytes.

**Figure 8.**
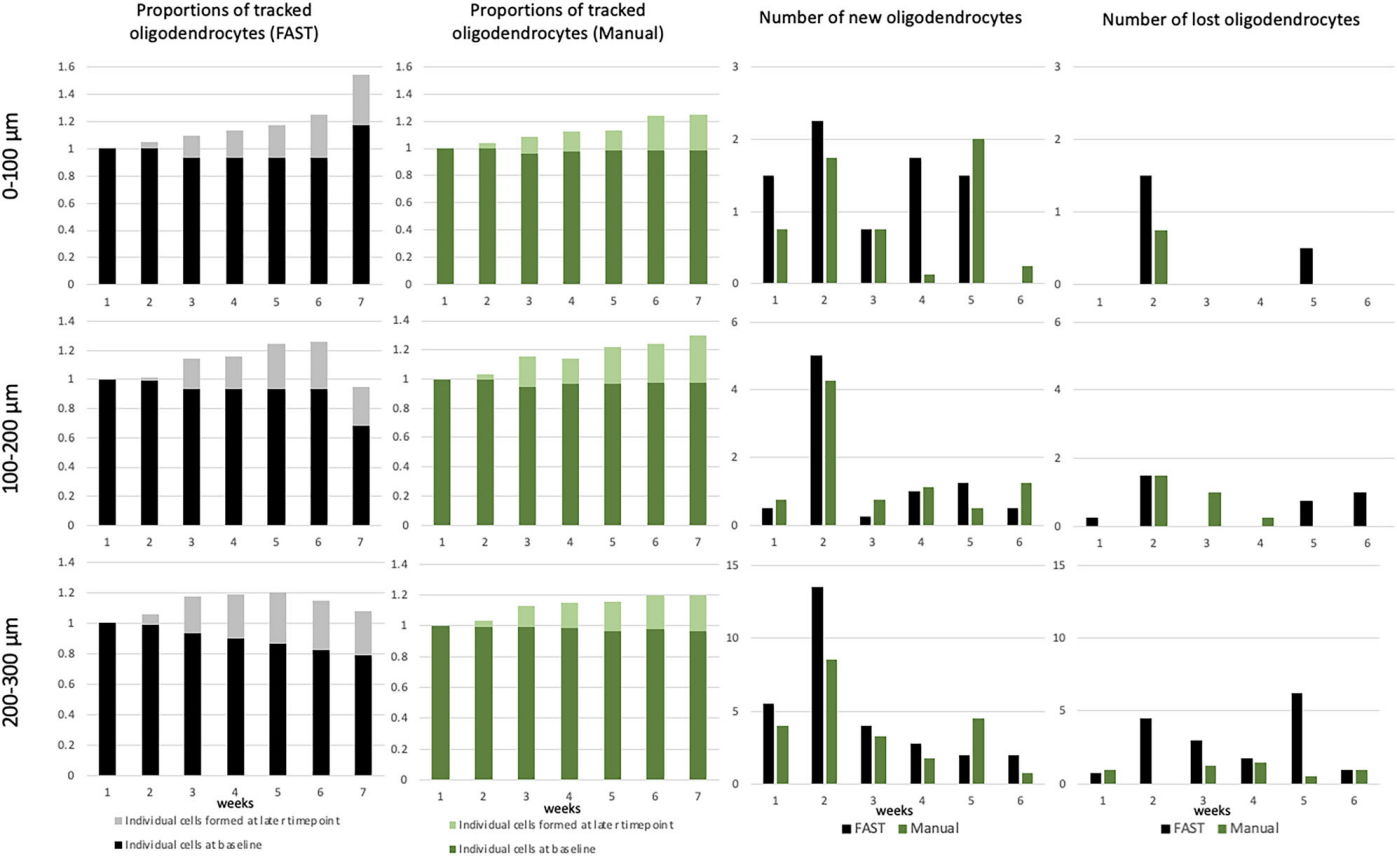
Result of the FAST model across timepoints, stratified by depth in somatosensory cortex for control mice (*n* = 2 mice). The rows indicate the 0 − 100 μm, 100 − 200 μm, and 200 − 300 μm depth regions of the images. The first column shows the average number of tracked oligodendrocytes by the FAST model. The second column shows the average number of tracked oligodendrocytes by blinded human observer. The third column shows the average number of newly formed oligodendrocytes. The fourth column shows the average number of lost oligodendrocytes.

We next assessed the performance of the FAST model by stratifying by depth of superficial somatosensory cortex in increments of 100 μm from the pial surface as previously analyzed in Orthmann-Murphy et al. (2020). The proportions of the average of tracked total oligodendrocytes per volume, as well as the count of new/lost oligodendrocytes at time-points after baseline imaging, as annotated by the FAST model and blinded human observer, stratified by depth, are presented in Figure 7 for cuprizone-treated mice (5 image sets from *n* = 3 mice ) and in Figure 8 for control mice (3 images sets from *n* = 2 mice).

For images from cuprizone-treated mice, the FAST model over-annotated new/lost oligodendrocytes in 0 − 200 μm zones, the superficial region with relatively fewer oligodendrocytes (Hughes et al., 2018) and under-annotated new/lost oligodendrocytes in 200 − 300 μm zones, where density of oligodendrocytes is higher (Hughes et al., 2018), compared to the blinded human observer. However, the difference was minimal. In the 0 − 100 μm zone (superficial), the estimated regression coefficient of the FAST model on blinded human observer was 1.02 (95% CI: 0.82 to 1.15). In the 100 − 200 μm zone, the estimated regression coefficient of the FAST model on blinded human observer was 1.12 (95% CI: 0.92 to 1.32). In the 200 − 300 μm zone, the estimated regression coefficient of the FAST model on blinded human observer was 1.30 (95% CI: 1.03–1.58). In the 0 − 100 μm zones, the mean absolute difference of the number of annotated new oligodendrocytes was 0.59 and the mean absolute difference of the number of annotated lost oligodendrocytes was 0.58. In the 100 − 200 μm zones, the mean absolute difference of the number of annotated new oligodendrocytes was 0.56 and the mean absolute difference of the number of annotated lost oligodendrocytes was 0.58. In the 200 − 300 μm zones, the mean absolute difference of the number of annotated new oligodendrocytes was 1.07 and the mean absolute difference of the number of annotated lost oligodendrocytes was 1.72.

For images from control mice, the FAST model over-annotated new/lost oligodendrocytes in almost all zones compared to the blinded human observer, but the difference was minimal. In the 0 − 100 μm zones, the estimated regression coefficient of the FAST model on blinded human observer was 1.06 (95% CI: 0.98 to 1.13). In the 100 − 200 μm zones, the estimated regression coefficient of the FAST model on blinded human observer was 0.92 (95% CI: 0.65 to 1.18). In the 200 − 300 μm zones, the estimated regression coefficient of the FAST model on blinded human observer was 1.23 (95% CI: 0.79–1.68). In the 0 − 100 μm zones, the mean absolute difference of the number of annotated new oligodendrocytes was 0.60 and the mean absolute difference of the number of annotated lost oligodendrocytes was 0.21. In the 100 − 200 μm zones, the mean absolute difference of the number of annotated new oligodendrocytes was 0.52 and the mean absolute difference of the number of annotated lost oligodendrocytes was 0.54. In the 200 − 300 μm zones, the mean absolute difference of the number of annotated new oligodendrocytes was 2 and the mean absolute difference of the number of annotated lost oligodendrocytes was 2.08.

### FAST model annotation applied to astrocytes across timepoints

While our method so far centers around segmenting oligodendrocytes, this can be extended to study other types of cells studied by longitudinal *in vivo* imaging. For example, astrocytes are complex cells distributed throughout the central nervous system (CNS), with highly branched processes that interact with all other CNS cells (Khakh and Deneen, 2019). Astrocytes have many important functions in development and the healthy adult CNS, but also respond and contribute to CNS damage (Escartin et al., 2021). To demonstrate the applicability of the FAST model to astrocytes, we applied the FAST model to a set of similarly acquired four-dimensional images from transgenic mice expressing TdTomato in the cytoplasm of astrocytes and GFP in oligodendrocytes (GLAST-CreER; R26-lsl-tdTomato; MOBP-egfp) and annotated the astrocytes over time. We used the same parameters as above for oligodendrocyte annotation. The total number of astrocytes, as well as the number of new and lost astrocytes were annotated.

**Figure 9.**
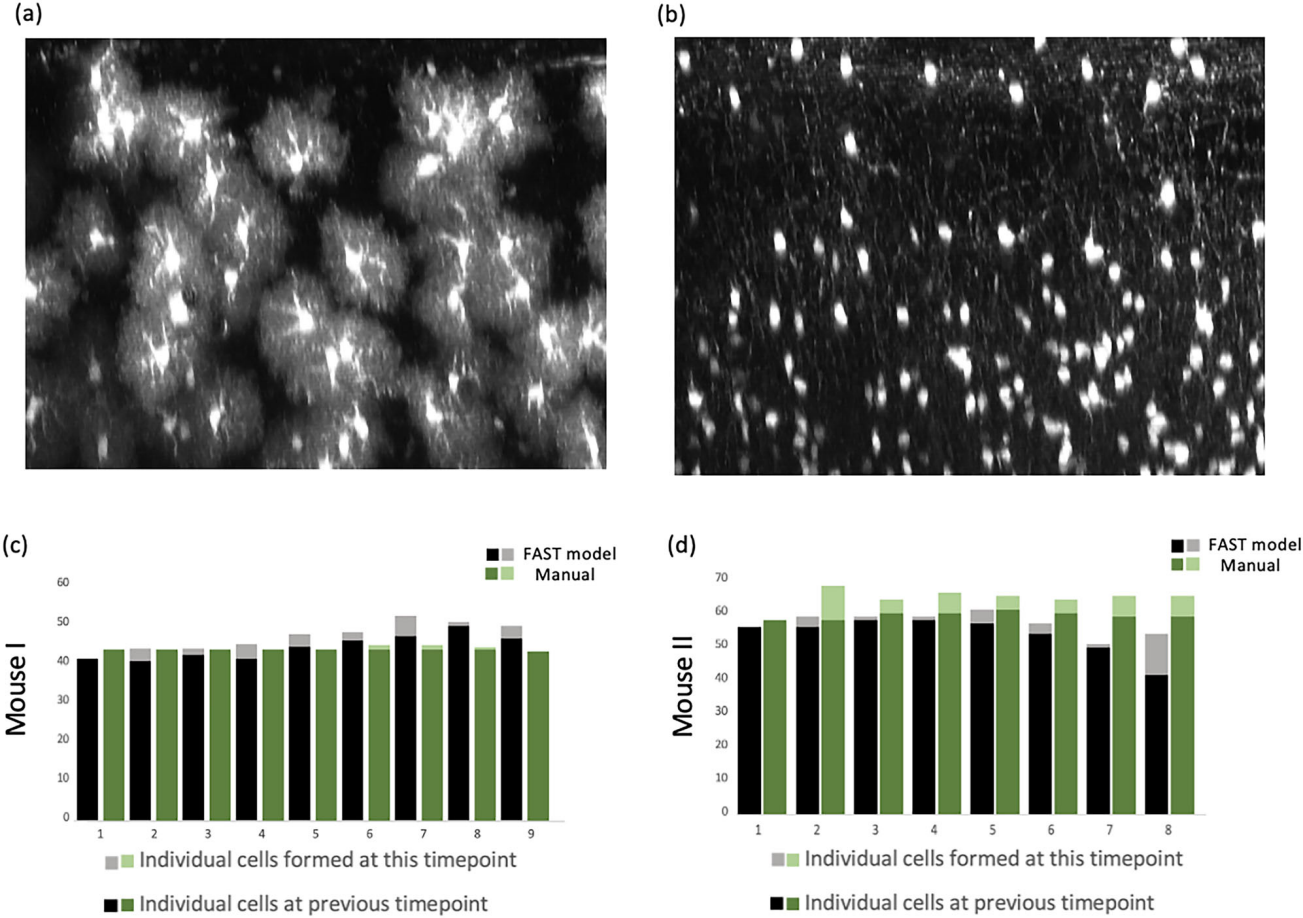
Result of the FAST model on astrocyte annotation. ***a***, An example of a cortical region containing TdTomato+ astrocytes, shown as a maximum intensity Y-projection, 405 μm × 300 μm) ***b***, The same cortical region as in (*a*), but the corresponding location of GFP+ oligodendrocytes (coronal view, 405 μm ×300 μm × 425 μm). ***c*,*d***, Results of the FAST model applied to astrocyte annotation (*a*) across time-points. The number of tracked astrocytes across time-points, annotated at baseline/previous time-point (by the FAST model in black, by the blinded human observer in green) and each later time-point (by the FAST model in light grey, by the blinded human observer in light green) is presented.

**Figure 10.**
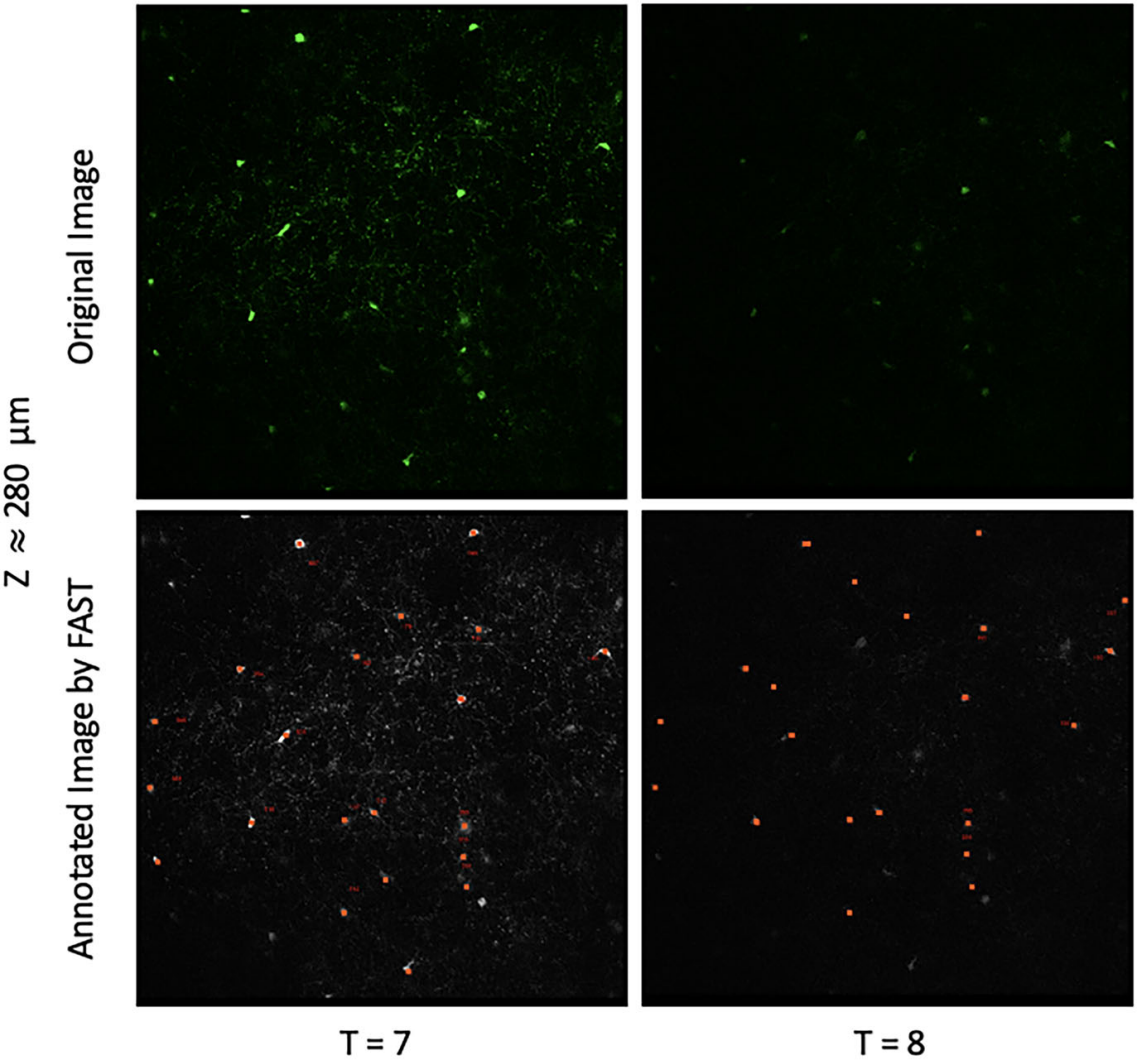
Examples of timepoints from control MOBP-egfp mouse with a low-signal-noise final time-point. Original images in the first row and annotated images by the FAST model in the second row. First and second columns show the original/annotated image at *T* = 7 (normal) and 8 (under-exposed). The index number next to the red rectangular is the unique identifier that given by the FAST model. Red rectangles with index numbers in the annotated image indicate that oligodendrocytes are annotated at both the seventh and eighth timepoints. Red rectangles without index numbers indicate that oligodendrocytes are annotated at the seventh time-point but lost at eighth timepoint by the FAST model. The original image at T=8 has low signal-to-noise and the FAST model did not annotate the oligodendrocytes (index numbers exist at *T* = 7 but not at *T* = 8) and therefore reports many lost oligodendrocytes.

The result of the FAST model is presented in Figure 9 and the images with the FAST-annotation as well as the spreadsheet that reports the coordinates of cell bodies, unique cell identifier, and the size of the cell bodies can be found at: https://drive.google.com/drive/u/2/folders/1r49L1ktZlrp4eW96CfNXQtaWaRT9Bjfw. The FAST model annotated astrocytes similarly to the blinded human observer (mean absolute difference: 5.2). The FAST model over-annotated the new/lost cells at a later time-point in Mouse II and we confirmed that this was due to differences in signal-to-noise ratio of acquired tdTomato signal at *T* = 7 and *T* = 8. As the FAST model was initially developed to identify dying oligodendrocytes as exhibiting decreasing intensity and new cells with increasing intensity (Fig. 10), the FAST model over-reported loss of cells at *T* = 7 and gain of cells at *T* = 8, where signal-to-noise was greater. By comparison, manual annotation detected loss of only one astrocyte at *T* = 7 and gain of one astrocyte at *T* = 8. Overall, this result shows that the FAST model can be extended to annotate other cell types with default FAST model parameters, but distinct biological parameters unique to the cell being tracked offer the opportunity to modify these parameters. For instance, we can consider using more lenient parameters to segment astrocytes, which have distinct processes from myelin sheaths. By choosing the candidate voxels with intensity values up to 98th percentile (instead of 99th percentile) in the preprocessing step, and setting the 70th percentile of intensity value as a threshold (instead of 80th percentile) to label the ROI and background when the two beta distributions are not found, the mean absolute difference of astrocyte annotation between the FAST model and the blinded human observer improved to 0.9 (5.2 → 4.3).

### Verification of FAST cell fate output

We confirmed the FAST-determined fates of individual FAST-identified cells (637 total cells from four individual datasets [three control data sets, each with 160, 170 and 187 FAST-labeled cells; and 1 cuprizone-treated data set, with 120 FAST-labeled cells). We used custom MATLAB code to visualize cropped stacks centered around each FAST-labeled cell to review the presence or absence of each cell at each time-point. The added time of this partially automated visual verification step ranged from <1 − 30 s per cell. For most cells (365 cells with all time-points accounted for by FAST-labels) review time was generally ≤5 s. For the remaining cells, the average review time per cell was around 30 s because they had at least one time-point that was unlabeled by FAST.

**Figure 11.**
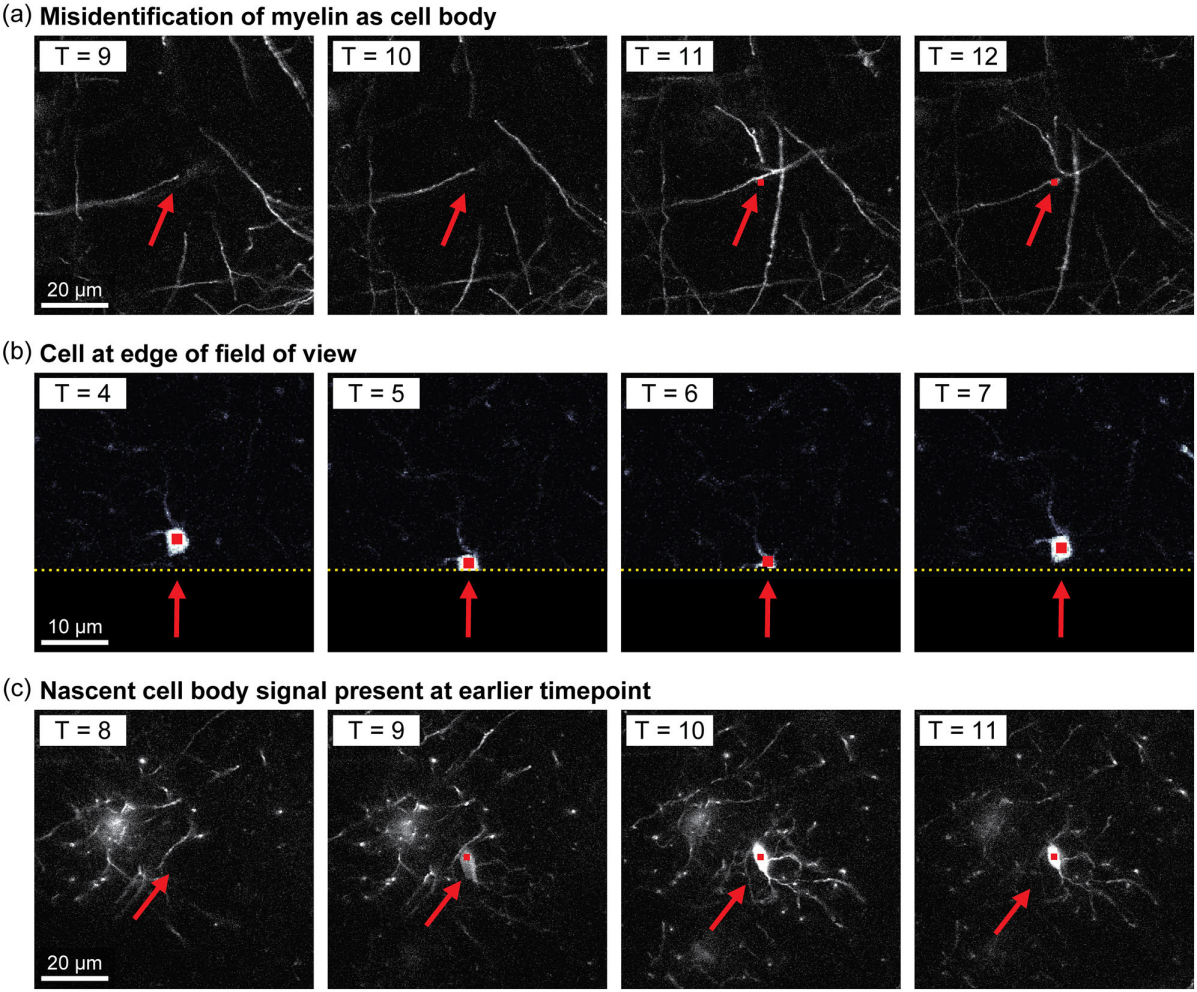
Partially automated visual verification of FAST-labeled cells : ***a***, An example of FAST mislabeling a myelin sheath structure (arrow) as an oligodendrocyte cell body at time-point 12 (*T* = 12). Red squares represent where FAST identified a soma (*T* = 12), or where a soma would be observed in the subsequent time point (*T* = 11) ***b***, An example of an oligodendrocyte cell body located at the edge of the X-Y field of view (arrow, *T* = 4). This cell left the field of view at *T* = 6 and returned at *T* = 7. Yellow dashed line represents an edge of imaged region. ***c***, An example of a faint (nascent) GFP signal (arrow, *T* = 9), indicating the presence of a newly-forming oligodendrocyte cell body that was detected at *T* = 10 onward. (a,c) Scale bars =20 μm, (*b*) scale bar =10 μm.

We found that FAST rarely misidentified other structures (typically myelin sheaths) as oligodendrocyte cell bodies (22 instances; an error rate of 3.45%, see example in Fig. 11*A*). In some cases, FAST identified cells as lost for at least one time-point because they were at the edge of the field of view (62/637 cells at the Z-depth limit; 43/637 cells at the X-Y field of view edge; see example in Fig. 11*B*). FAST rarely incorrectly relabeled stable cells as new cells at later timepoints (3 cells in all four datasets). For the remaining cells with missing timepoints, we confirmed that FAST correctly accounted for intermediary timepoints with low-signal-noise and labeled cells as stable (data not shown). With this verification step, we were also able to quantify the presence of nascent (faint) GFP signal of newly forming oligodendrocytes (Fig. 11*C*), because FAST automatically labels cell position (in X,Y,Z) at the time-point prior to emergence of new cells (13/164 new cells formed after the baseline time-point). Together, we were able to perform rapid post hoc confirmation that was only possible due to the segmentation and tracking provided by the FAST model.

## Discussion

We propose an unsupervised, fast, and accurate oligodendrocyte segmentation system that segments and tracks oligodendrocytes in longitudinal three-dimensional images with minimal human input. The FAST model adjusts for the heterogeneous brightness of fluorescence signal across cortical depth and removes cell processes like myelin sheaths and noise in order to segment the cell bodies of oligodendrocytes. By using CCL, the FAST model segments and tracks the fate of oligodendrocytes with volumetric quantification.

The FAST model was developed with no training step, taking 25–30 min per image set. This achievement is crucial as manual cell segmentation can lead to long delays in analysis. For the blinded human observers in our study, it took from 4 to ten hours to manually annotate and track individual oligodendrocytes in a single image set. An alternative supervised method such as convolutional neural network (CNN) that requires a fully annotated training set as well as model tuning would take additional steps and time. As the FAST model was developed as an unsupervised method, we expect our FAST model can solve this analysis bottleneck. In addition, unlike deep learning technologies which have been criticized for its black-box nature, the FAST model allows a user to understand each step and can therefore tune parameters (as we showed for astrocyte annotation). Given the nature of unsupervised methods where the training data is not needed, the FAST model is not error-free. However, the first pass using the FAST model could save a significant amount of time in the analysis pipeline.

To further enhance accuracy and efficiency, we incorporated a partially automated visual verification step, which allows users to modify and confirm the FAST model output. This verification process enables users to remove cells located at the edges of the field of view, where cell presence and absence are less reliable due to potential imaging limitations. Additionally, this step facilitates the detection of subtle signals, such as the nascent GFP signal observed in emerging oligodendrocytes, which might otherwise be overlooked. By integrating this verification step, we support novel biological discovery while keeping the manual correction time minimal, typically under one hour per dataset. This approach provides a robust yet time-effective method to refine segmentation output and ensure accurate data representation in longitudinal studies. For this reason, we recommend users to employ the model as a first step for segmentation. Manual segmentation confirmation/correction from the output of the FAST model requires less than an hour per one image set.

**Figure 12.**
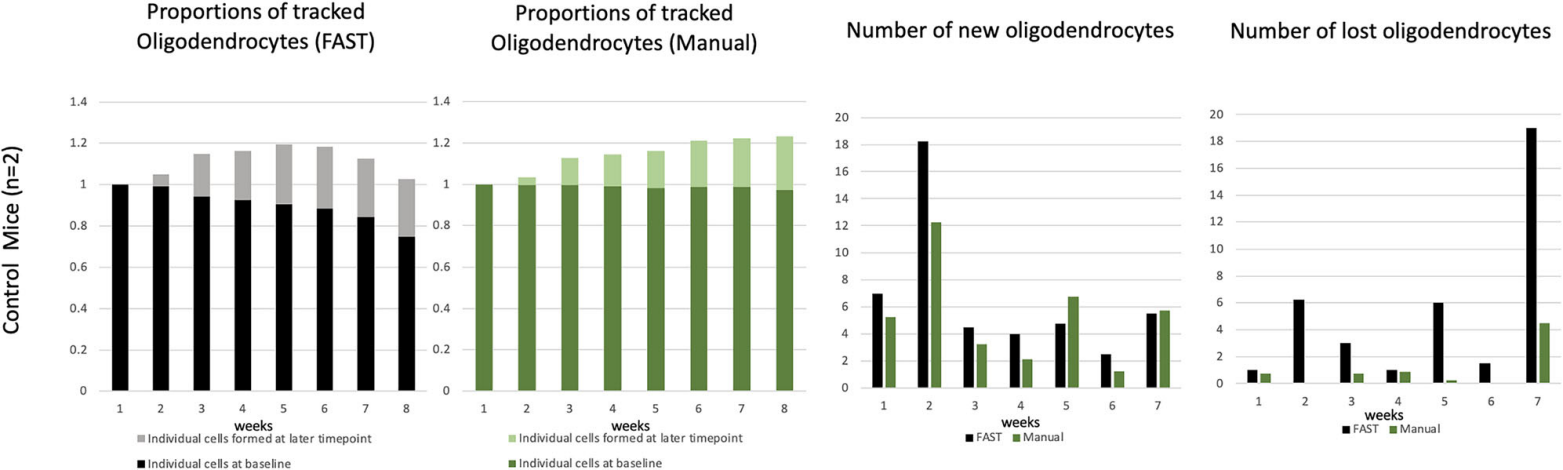
Results of the FAST model across timepoints when applied to the control mice including the eighth time-point image set with reduced signal-to-noise. The first/second column shows the proportions of the average number of tracked oligodendrocytes by the FAST model/blinded human observer. The third column shows the average number of newly formed oligodendrocytes at each time-point. Each time-point represents the number of new oligodendrocytes observed at its next time-point. The fourth column shows the average number of lost oligodendrocytes at each time-point. Each time-point represents the number of lost oligodendrocytes observed at its next time-point. The FAST model reports notable loss of oligodendrocytes at seventh time-point, which was not observed by manual annotation, so this time-point was rejected for analysis.

The FAST model showed unsatisfactory performance when applied to the control mouse data set that included image timepoints with lower signal-to-noise (Fig. 12). Based on manual tracking, we expected to see a stable number of baseline oligodendrocytes, and the addition of new oligodendrocytes at later timepoints (Hill et al., 2018; Hughes et al., 2018; Orthmann-Murphy et al., 2020). However, the FAST model reported loss of oligodendrocytes at a time-point with low signal-to-noise of the GFP signal. This was due to the variability of image quality intrinsic to longitudinal *in vivo* imaging, including varying dural thickness over weeks (Holtmaat et al., 2009). The first row of Figure 10 shows the one of original images that represent the varying image acquisition parameters. At *T* = 8, the GFP signal detected from oligodendrocytes is low compared to prior time-points when acquired at the same settings. As a result, the FAST model reported marked loss of cells at *T* = 7 (fourth column) and a drop in the number of tracked oligodendrocytes at *T* = 8 (first column) as shown in Figure 12. In this regard, control images can be used to test quality of fluorescence detection for each individual time-point, and allow rejection of images with insufficient signal-to-noise for analysis. Our FAST model can also be used to detect any irregularities that arise over longitudinal experiments–comparison to prior images may prompt immediate assessment of image acquisition parameters in real time. Alternately, FAST model code can potentially be modified to correct for differences in signal-to-noise ratio at individual time-points. There is no general rule for rejecting images. However, we suggest carefully considering the initial output from the FAST model and checking whether the underlying conditions necessary for successful implementation of the FAST model are met. Before rejecting images, users should attempt to refine the model parameters and check whether the FAST model can be improved, as demonstrated in astrocyte annotation.

The FAST model assumes that the images have been processed to be spatially registered longitudinally, as the CCL annotates the oligodendrocytes based on the location. If images are misaligned, which may occur due to 3-dimensional movement of cells within the brain itself (Orthmann-Murphy et al., 2020) then the CCL fails to recognize oligodendrocytes that exist over time and instead reports false loss of oligodendrocytes and later formation of new oligodendrocytes. Due to misalignment in some cases, FAST reported a similar number of tracked oligodendrocytes to blinded human observers but reported higher numbers of new/lost oligodendrocytes. In the future, extensions of the FAST model to incorporate automated longitudinal alignment in the tracking algorithm could aid the FAST model to align the images accordingly. For now, the FAST model depends on the FIJI plugin “Correct 3D Drift” for image alignment. We believe that a better algorithm can further improve longitudinal three-dimensional image alignment. We acknowledge that having 5 sets of images from three cuprizone-treated mice and 3 sets of images from two control mice to quantify oligodendrocytes limits generalizability of our results. However, the addition of 3 sets of images from two control mice to quantify astrocytes suggests that FAST can be applied two four dimensional stacks and multiple settings and cell types. We limited our analysis here to image stacks acquired at the same resolution using the same objective. Future studies will focus on increasing sample sizes and images acquired using additional water immersion objectives (including 10x objectives) for more robust validation and our ongoing experience will be shared publicly at https://github.com/PennSIVE/bossr.

Cell segmentation and quantification is a crucial part of the analysis of two-photon microscopy data, but was a significant barrier because manual curation by blinded human observers is costly, time-consuming, and not scalable to large datasets. Here, we propose a model that allows automated quantification that can give robust, reliable, and reproducible results. We expect our FAST model to be able to solve an important bottleneck in cell imaging research as our model does not require fully curated data (training data), reduces computational time and costs, and most importantly recapitulates cell dynamic patterns as measured by a blinded human observer. Our work is a significant step forward in automated segmentation and tracking of ROI in longitudinal three-dimensional microscopy images, and we expect the FAST model to facilitate research in this area.

## Synthesis

Reviewing Editor: Lindsay De Biase, University of California Los Angeles Decisions are customarily a result of the Reviewing Editor and the peer reviewers coming together and discussing their recommendations until a consensus is reached. When revisions are invited, a fact-based synthesis statement explaining their decision and outlining what is needed to prepare a revision will be listed below. The following reviewer(s) agreed to reveal their identity: Ania Majewska.

This manuscript presents a new open-source tool for automated tracking of oligodendrocyte cell bodies (and potentially other cell types) longitudinally within 3D images. Both reviewers agreed that automated analyses are needed to deal with large data sets being created with new imaging techniques and this tool could potentially save valuable experimenter time in the specific analysis of in vivo cell death and regeneration. However, the reviewers also had key concerns that need to be addressed.

Major Concerns:

1) Gauging by the quantification presented in Figs 3-5, supplemental data, and example images in S4, the FAST model does not appear to accurately reflect ground truth that is obvious to the human eye. The numbers of new and lost oligodendrocytes differ quite a lot between FAST vs. manual quantification, and when the absolute numbers are so low, these differences amount to a significant discrepancy. Although the authors categorize FAST errors as 'minimal', i.e. mean differences of 1.83-2.69 oligodendrocytes, these numbers do not seem minimal when the total number of lost and gained oligodendrocytes fluctuate between 1 and 12 in most time points. In image S4, setting aside the decrease in image intensity at T8, there are obvious errors made by the algorithm that account for a large percentage of the total numbers of oligodendrocytes present in the field of view. Given these discrepancies, it seems that manual verification and correction will be critical for experiments where the expected effect size is less than 30-40%. Therefore, it is unclear how much time can be saved by the application of FAST tracking on longitudinal imaging datasets. To address these concerns the authors should address the following:

1a) An attempt should be made to quantify the % error introduced by the FAST model over human observers. This should be done for a full experiment, i.e. graph data by image, and then by mice, quantified by FAST vs. by eye. Statistics should compare between experimental groups and between quantification methods.

1b) The authors use a metric called a BA plot to assess agreement between FAST and human observe generated data. It is not immediately clear what this metric assesses, nor is it clear how one should interpret this sentence: "BA plots (in the first row) show the good agreement between the FAST model and the blinded human observer as almost all data points are within the 1.96 times the standard deviance of the differences." It is difficult to contextualize what 1.96 times the standard deviance (deviation?) means, and what a 'good' standard deviance would be. The authors should make clear why 'almost all data points are within the 1.96 times the standard deviance' is a desirable metric.

1c) Given the large amount of error apparently introduced by FAST, manual verification/curation appears to be required for accurate image analysis, though perhaps a first pass using FAST could save time overall in the analysis pipeline. The manuscript would benefit from a proper quantification of time spent for analysis per image, with and without FAST, to demonstrate the value of applying FAST.

1d) Related to the above point, the goal for most experimenters is for the analysis to reflect ground truth, which means that images annotated by FAST will require additional manual curation. The authors would significantly lower the threshold for adaptability by presenting a full analysis pipeline that incorporates FAST as a first step pass that would be followed by clear instructions for manual adjustments. Estimates of time spent per image should incorporate time spent on manual corrections.

2) The authors need to add details regarding IACUC approval and details on animals used for generating image datasets. Specifically, in the methods please create an animals section and move the information on animal usage from "Image Acquisition and Preprocessing" to this new section and provide IACUC assurances and more information on housing, treatment, anesthesia etc.

3) The sample size - n=2 for control and n=3 for cuprizone treatment - is low. A higher n is required for a more robust comparison between the model and blinded observer tracking and allowing for adequate statistical comparisons between data generated from FAST and data generated from blinded human observers.

4) Can the authors give example images where "two beta distributions are fit, set the intersection point as a threshold to label the region of interest (ROI) and background" versus "only one beta mixture component is detected, then voxels under a specified threshold are labeled as background, and those above the threshold are labeled as cell bodies" (Figure 2, the Yes or No boxes). I assume that the later scenario is likely coming from an image with low signal-to-noise ratio or when the intensity of processes doesn't differ much from that of the cell bodies? Also did the author see under- or over-annotation problem mostly coming from the percentile threshold, or equally distributed between the two ways?

5) There are multiple formatting errors throughout the manuscript, please make sure to proofread carefully.

Minor comments:

1) Please reword and or clarify this part of the methods on CCL as I had a hard time understanding it: "To adjust the heterogeneous resolution of the interim image, we assumed that the subgraph located at the neighborhood of {i, j, k} over time-point t to be considered as the same subgraph This kernel of the neighborhood can be optimized. Once G is determined, we filtered out the connected components in G that have the node sets with less than 30 nodes."

2)As the authors mentioned, the lower signal to noise ratio in later time points and deeper cortex negatively impacts the accuracy of the model. To optimize the model, can the authors suggest a built-in way to either drop those images or enter a different threshold?

3) The authors also acknowledge that one limitation of CCL is that across time the brain will morph and that will impact the accuracy of tracking annotated cells. Can the authors expand on this "extensions of the FAST model to incorporate automated longitudinal alignment in the tracking algorithm could aid the FAST model to align the images accordingly." in the discussion with potential FIJI plug-ins or algorithms to be implemented?

4) No figure title for Fig. 1, 4, 5. Please add these.

5) I wonder whether there is a cell body size filtering step post-median filtering prior to CCL? Could the authors clarify this point?

6) Figure S4 left bottom panel title reads 'Automated Image by BOSS'
